# Re-conceptualizing sustainable urban sanitation in Uganda: why the roots of ‘Slumification’ must be dealt with

**DOI:** 10.1186/s12889-021-11029-8

**Published:** 2021-05-26

**Authors:** Japheth Nkiriyehe Kwiringira, Robert Kabumbuli, Henry Zakumumpa, James Mugisha, Mathias Akugizibwe, Paulino Ariho, Joseph Rujumba

**Affiliations:** 1 Department of Sociology and Social Administration, Kyambogo University, Kampala, Uganda; 2 Department of Sociology and Anthropology, Makerere University, Kampala, Uganda; 3 College of Health Sciences, School of Public Health, Makerere University, Kampala, Uganda; 4 Department of Pediatrics, College of Health Sciences, Makerere University, Kampala, Uganda

**Keywords:** Urbanization, Urban livelihoods, ‘Slumification/slumify’, Urban poverty, Sustainable urban sanitation, Uganda

## Abstract

**Background:**

Country-wide urbanization in Uganda has continued amidst institutional challenges. Previous interventions in the water and sanitation sector have not addressed the underlying issues of a poorly managed urbanization processes. Poor urbanisation is linked to low productivity, urban poverty, unemployment, limited capacity to plan and offer basic services as well as a failure to enforce urban standards.

**Methods:**

This ethnographic study was carried out in three urban centres of Gulu, Mbarara and Kampala. We explored relationships between urban livelihoods and sustainable urban sanitation, using the *economic sociology of urban sanitation framework.* This framework locates the urbanization narrative within a complex system entailing demand, supply, access, use and sustainability of slum sanitation. We used both inductive and deductive thematic analysis.

**Results:**

More than any other city in Uganda, Kampala was plagued with poor sanitation services characterized by a mismatch between demand and the available capacity for service provision. Poor slum sanitation was driven by; the need to escape rural poverty through urban migration, urban governance deficits, corruption and the survival imperative, poor service delivery and lack of capacity, pervasive (urban) informality, lack of standards: *‘to whom it may concern’* attitudes and the normalization of risk as a way of life. Amidst a general lack of affordability, there was a critical lack of *public good* conscience. Most urbanites were trapped in poverty, whereby economic survival trumped for the need for meeting desirable sanitation standards.

**Conclusions:**

Providing sustainable urban livelihoods and meeting sanitation demands is nested within sustainable livelihoods. Previous interventions have labored to fix the sanitation problem in slums without considering the drivers of this problem. Sustainable urban livelihoods are critical in reducing slums, improving slum living and curtailing the onset of *slumification*. Urban authorities need to make urban centres economically vibrant as an integral strategy for attaining better sanitation standards.

**Supplementary Information:**

The online version contains supplementary material available at 10.1186/s12889-021-11029-8.

## Background

Poor sanitation is a major indicator of urban poverty and poor health [1, 2]. Squatter settlements that are common in urban areas are frequently attributed to poverty [3, 4]. Compared with their high-income counterparts, low-income countries are disproportionately affected by slum settlements [1, 4–6] . In addition, urban areas in the global south are associated with higher poverty [7, 8]. The high rate of urban poverty in Africa has not reduced the region’s rate of urbanization [4]. In fact, the growing urbanization is partly attributable to poor economic performance and bad governance in rural areas [9, 10]. Post-independence Uganda has been characterized by declining economic growth, low productivity, conflict [11, 12] and governance deficits. The later have negatively impacted urbanization [13, 14] coupled with poor urban service delivery [15]. Admittedly, while the colonial planning frameworks [16] had their weaknesses and biases replete with racism, the post-colonial institutional decay [17] further accelerated the collapse of the economy and increased the national debt with the resultant collapse of urban services including sanitation.

Urbanization involves populations moving from rural to urban centres and the re-organizing of activities that lead to permanent urban residence [18]. Sustainable urbanization continues to present a major challenge to urban authorities across the world [19, 20]. In Sub-Saharan Africa, post-independence urbanization has been a haphazard process that has exacerbated urban poverty [1, 2, 21–24]. Urbanization in Uganda has continued unabated due to well-known push and pull factors [22, 25] amidst economic stagnation, inequality and urban decay [26, 27]. This context, continuously makes most urban residences unsuitable for human habitation [28] due to congestion, poor housing, poor service delivery including water, sanitation and hygiene [29, 30]. Urbanization in itself is a progressive process as was the case in the first city states of Sparta, Mesopotamia and Athens, that were centers of civilization and *thinking*, high productivity and innovation [31–33]. Cities have been hotbeds of democracy, good governance [33, 34] and better infrastructure [35]. Good urban services tend to enhance the progress of communities [36, 37]. Such is the outlook to the city as an economy and polity [38, 39]. This appears to be a missing in Uganda’s urbanizing agenda. Urban sanitation conditions in Uganda are deplorable [24, 40, 41]. Sanitation choices available to the urban poor are limited onsite facilities that are shared by up to 30 individuals or 7 households, with private sanitation being a privilege for a few house owners [40]. Studies have shown that, shared sanitation facilities in slums are abandoned after a short time [29, 42]. This is made worse by the fact that the cost of an improved sanitation facility exceeds the annual per capita income of many slum dwellers [40].

### An outlook at Uganda’ urbanization

In Uganda, 24.36% of the total population lives in cities and urban areas with a population growth rate of 3.3% [43]. Kampala is Uganda’s oldest city and constitutes about 25% of the country’s total urban population. In Uganda, about 60% of the urban population (representing about 18% of the total population) live in slums and informal settlements [43], with pervasive unemployment and congestion [44]. Both individual and institutional factors sustain the slum experience in Uganda. Kampala city is the most affected by unplanned and unsustainable urbanization. Mbarara city, the biggest (administrative and business) urban centre and transport hub in the western region has been under pressure from rural populations that migrate to seek employment to escape low wages and poor performance of rural agriculture. The urbanization of Gulu [45, 46] was partly driven by people fleeing the two decade brutal civil war between the Government of Uganda and the rebels of the Lord’s Resistance Army -LRA [47, 48]. For northern Uganda, the humanitarian crisis [11, 49] resulting from this conflict had long- lasting impressions on the urbanization of Gulu which continues to host those that seek an *urban dividend* [50, 51]. Gulu town has also been host to those fleeing conflict from neighboring Sudan, South Sudan, Eritrea and Somalia as well as those affected by hunger in Northern Uganda [52]. Overall, lack of a robust industrial sector and employment opportunities have had negative consequences that partly explain the persistence of slum conditions [53].

### Theoretical framework

Drawing on the *economic sociology* [54–56] of urban sanitation, we explored relationships between urban livelihoods and sustainable urban sanitation in Uganda. Economic sociology relates to how material conditions of life are produced; and reproduced through social processes, including the production, supply, demand, and consumption goods and services [57], structure and dynamics [58]. There have been no studies exploring the linkages between unsustainable urban sanitation and unsustainable urban livelihoods.

## Methods

### Study design and setting

An ethnographic [59, 60] study was conducted in Gulu (Bar-Dege and Pabo zones), Mbarara (Mandela and Kiyanja zones) and Kampala (Ddobi and Kisasiizi zones). Study sites were purposively selected through reconnaissance and ranking by Key informants. Kampala is the largest and national capital with the most slums. Mbarara was selected on account of being the regional capital in the western region as well as having the largest number of slums after Gulu. Participants were the urban poor that had stayed in study areas for more than five (5) years. The inclusion criteria were that, participants should have been resident in the study area for not less than 60 months and had adequate knowledge of the area dynamics and processes. Data were collected through Focus Group Discussions (FGDs), Key Informant Interviews (KIIs), in-depth Interviews (IDIs) and community meetings. We developed research instruments for this study (see attached supplementary file 1). Only adults (18 years and above) were involved in the study after obtaining written informed consent [61, 62]. Key informants representing urban sector players were drawn from Uganda Government Ministries, Departments and Agencies-MDAs (central and local), academia, civil society and the private sector. This approach helped in achieving diversity (see Table 1) of perspective but also to validate study findings. Local languages (Luganda and Swahili for Kampala; Luo for Acholi and Runyankore/ Rukiga for Mbarara) were used to interview participants as a means to obtain the intended meaning. These interviews were then translated into English. Data were collected by graduate researchers with vast experience in qualitative research; assisted by AM and the first author who is an urban and public health sociologist. On average, FGDs (of about 10 persons on average) lasted 1 h, KIIs took about 45 min and IDIs 1 h 15 min. We conducted an extensive review of literature on urban sociology, demography, populations studies, urban planning, water, sanitation and hygiene; urban geography and public health.

**Table 1:** Summary of sample, methods study participants (*n* = 156)

Category of participants	Number
KII 9 taken from each study site (Academia; Government Ministries, Departments and Agencies -MDAs, Local government: Technical and political leaders)	018
IDIs (Private sector property developers/landlords and Civil society NGOs/CBOs) -2 from each sire	006
Community /windscreen transects −1 per study site	*003*^a^
Slum Resident	
i) FGDs (Women, men and youth) for each zone. Discussed urban sanitation experience, prevailing sanitation situation, challenges, options, coping and future plans	18 groups 126 participants
ii) Community meetings −2 taken from each site	006

^a^*This is a method without participants. Therefore, the 3 transects are not included in the number of study participants*

### Summary of data sources

#### Data analysis

At the end of each field day, the research team converged to reflect on the emerging study findings. This formed the basis for further discussion in form of developing themes for preliminary analysis. After data collection, a detailed debrief was held with the entire field team as a means to further reflect and share insights, patterns and themes that had emerged. Interview transcripts were first read by the first author who grouped them by themes. These themes were then shared among the research team who compared the English version with the local languages used at data collection. After this, harmonized themes were jointly reviewed to ensure consistency. The next stage was to extract and refine what was input into NVivo 11 for windows for software assisted analysis. We used both inductive and deductive thematic analysis, where data were analyzed in an iterative manner [63] starting from reading the field notes and getting familiar with data which enabled the generation of codes as a form of preliminary analysis. The codes were further grouped into categories that generated seven (7) themes;

i) urbanization and escaping rural poverty through migration
ii) urban governance deficits
iii) corruption and the survival imperative
iv) poor service delivery and lack of capacity
v) pervasive (urban) informality: slums as poverty traps
vi) lack of standards ‘to whom it may concern’ and the normalization of risk as a way of life
vii) ‘slumification’ as a platform for poor urban sanitation.

The foregoing themes were then interpreted after a prolonged engagement with both the data; and the study team, where theory was relevantly deployed to make sense of the available data before synthesis, reporting and drawing of conclusions [64, 65]. Secondary literature was thematically analyzed using content analysis [66]. The study protocol was approved by the Institutional Review Board (IRB) at Makerere University School of Social Sciences (MAKSS REC 08.18.210) and the Uganda National Council of Science and technology –UNCST (UNCST SS273ES).

## Results

Study findings are presented on the basis of emerging themes as detailed in the following sections. From the derived themes, slums have a dual nature; both *as (i) physical locations* -space and a place and ii) *as a type of behavior or lifestyle*. Several factors encouraged and conditioned people to;

i) opt for slum settlements -slums as place due to poverty and restricted means.
ii) engage and position themselves into slum behaviours even when they could have done better.

While slums are informal places, not all informal locations are slums. Informality has to do with lack of planning and services and less with poverty which is a hallmark of slums. In this paper, we argue that ‘*Slumification’* relates to both informality and slum locations as both ‘place’ and *behaviours*.

### Urbanization and the need to escape rural poverty

A synthesis of data indicated that, the challenges of Uganda’s urbanization are a result of;

i) An attempt to escape rural poverty through migration and.
ii) once in the city, a determination to remain there owing to the perceived opportunities and better services. In-depth interview excerpts with a development expert highlight the extent of the problem.
  *‘The mere prospect of potential income together with the comparatively high concentration of social services in urban areas, with few rural options is a driver of the exodus from rural areas. Rural neglect is aggravated by government policies that don’t support value addition, industry exports and producer cooperatives that were once the heart of rural and regional economies. The perceived urban advantage is on account of the media which projects urban areas as places of bliss or at least having better life chances. This rural urban variation in perception and actual services fuels the rate of rural urban migration with more people being trapped into urban poverty, and seldom able to return to rural areas. Amidst this, urban centers do not have the capacity to deliver services including infrastructure provisions due to a low tax base. There are many generations of urban natives trapped in slums and these must stay put! The economy has few exports to be taxed to finance the delivery of social services. Once people do not have much taxable income, institutions can only do little...!’*

One government official noted that;

*‘Many farmers have been frustrated by poor agricultural support, low yields, low prices and failure to meet basic needs and yet they (farmers) have tried their best. This makes many people shun agriculture as livelihood and opt for the seemingly viable urban promises. Many people have left villages to come and ride motor cycles in Kampala.’*

The foregoing agrees with a think tank co-chair who noted that;

*‘Poor urbanisation cannot be delinked from a poor urban experience. An urbanization agenda that does not leverage productivity across the country will only create slums and not viable urbanisation. Unemployment cannot be delinked from urban poverty and poor service delivery including unsustainable urban services.’*

Participatory sessions indicated that, urban areas are an attraction for the young and those seeking a financial breakthrough. Having urbanized, participants were well aware of their plight, eager and worked around daily survival before improving their environs. That slum conditions were undesirable, was clear to participants. Slum dwellers also knew that they are not the cause of their predicament. They saw themselves as part of the urban configuration and would do better if the structural constraints were removed and more opportunity came their way. In Ddobi and Kisasiizi, FGD participants argued thus;

*‘We also love nice things. We know that we do not live-in suitable locations. This place [slums] exposes us to sickness, but what can we do?! Look at what we do; do we have jobs and income? We simply try to survive. when you are surviving, there are many things you do not think about, especially when you live from day to day and sometimes from meal to meal. Buying food and paying rent are the most critical issues. We know how to be presentable in public so that people can trust you … other issues like garbage collection, drainage and toilets; we are not yet at that level.’* Ddobi FGD

*‘Anyone that can survive year in and year out is a hero. To survival in this town is success! In such living conditions, we are not able to have some things right … maybe when our income increases then we can aspire for better things. Good houses and proper sanitation need better income...’ Even then, we are better off than being in the village where there’s no hope. At least, here when you see the rich you hope that somehow, you can get a chance to access their money … some rich people also started out like some of us … ’* Kisasiizi FGD

Such sentiments reflect the bitter-sweet experiences of most urbanites. In slums, hope, joy and pain were mingled in the prospect of *‘being like those better than oneself’.* This was put in perspective by recourse to popular culture. For example, the Ugandan music legend Philly Lutaaya in the hit song ‘*Jjangu tugende e-Kampala’* -literally translated, ‘come-on let us go to Kampala’ beckoned everyone to *‘come and go to Kampala*’ -the city. In this song, the city and urbanism were ‘a must’ for one to have a chance at scarce opportunities of life. From such popular culture, many people strive to have a *stint* in the city so as to enjoy a ‘good life’ and the perceived urban opportunities. It was reported that, at social functions especially in rural areas, Kampalans usually have their seats and food ‘reserved’ in addition to being especially recognized as city dwellers. This is what translated in the urban allure and mystique, attracting ‘non-urbanites’ in order to catch the *spice of life*. There were accounts of merely moving into the city and hoping things will work out -somehow, no matter how difficult things had become. In this sense, rural -urban migration became a means and hope to imagine the future.

### Urban governance deficits

Key informant data indicated that, the complexities of slum formation were deep-seated and include spoken and non-spoken, material and non-material aspects.

*‘While urban planning guidelines are laid down, the actual implementation of these guidelines is politically driven and not led by technical merits of sustainability. While such and similar actions do not create slums in a typical deprivation sense, the result of such actions is a slum environment that is not well serviced falling short of many urban planning canons. Most areas in Kampala have evolved and are not developed or planned. Almost all areas are at sub-optimal levels by global urban standards. If you look at most suburbs of Kampala where the affluent stay, they are rich men’s slums … they are areas of affluence but not planned.’* Urban planning and development professional working with Government Agency in Kampala.

Even ‘affluent’ neighborhoods lacked basic social services such as sewer lines, access roads, road reserves, leisure parks and walkways and yet, the residents had the means to afford better planned habitats. To an extent, this description suggests *a poverty* of welfare that is typical of slum life. Urban residents who were constrained by lack of means to survive took advantage of gaps in urban planning and enforcement to encroach on public spaces. On the other hand, urban authorities lacked the moral latitude and capacity to enforce compliance with urban standards. There was an ‘anti-planning agenda’ in the form of activism that thrived in slums, becoming part of the urban polity; a tool and source of power to resist law enforcement by urban authorities. We found that, urban governance canons had been enveloped by politicking and populism which did not work in public interest. There were notions of a private gain from available public goods.

### Corruption and the survival imperative

Urban commons especially open spaces like golf courses in most Ugandan planned towns (all planned towns are also pre-independence towns) had seen some sections degazetted to commercial use and expropriated to developers under the guise of *job creation.* What this demonstrates is a misunderstanding of *what it takes to have a functional city*. Findings from FGDs converged around a theme that, cities are places ‘*where the majority destitute and only few flourishes and get enriched … ’* Such perceptions among urbanites shaped their engagement with the city. Participants noted that, individual benefit is pursued almost *at all costs* with no regard to wider society and ‘publics.’ Many study participants had not seen much benefit from taxation and were reluctant to pay taxes. Such perceptions align with what has been documented in the form of lack of drugs in hospitals, poor roads and wider corruption. FGD participants expressed predatory sentiments regarding their strategy for staying in the city.

*‘In the city, you need to grab whatever you can as soon as you have the opportunity and retreat to private life...! you cannot change anything here … Government is not interested in the poor.’* FGD, Ddobi zone, city?

The above makes recourse to an extractive form of urbanization that is unsustainable. Both the urban and urbanizing populations were oriented into a culture of *no regard for general welfare* by encouraging ‘free riding’.

### Poor service delivery and lack of capacity

Findings point to a perceived conspiracy to plunder and misallocate public assets and resources. Participants argued that, corruption had become a form of culture that negatively affected the urbanization process. The situation was made worse by actors and stakeholders aiming at *quick gains* as opposed to dealing with root causes of the manifested situation, with little or no considerations of;

i) Root causes and drivers
ii) Conditions of the targeted population
iii) Manifested poor sanitation practices that need behavior change
iv) Why demand for sustainable sanitation is low
v) Sustainable urban livelihoods in delivering sustainable sanitation services.

*‘For the most part, there is no room for sustainable urban planning, and indeed urbanization as long as the available resources are deployed and exploited for private gain. At local government level, the concern is first and foremost local revenue mobilization and how to fund the budget. So long as revenue can be realized, the sustainability of the income source and the consequences of such actions are usually secondary... It is now fashionable to lose public space including wetlands and forest reserves, giving away of urban green spaces including golf courses to pseudo investors on the pretext that jobs will be created for the urbanizing population. This process of plundering public goods for short term gains and so-called quick gains is what gradually, but surely erodes regard for shared and public goods.* IDI with Urban management expert at an international development agency.

*‘We’re constrained! We mostly work on the basis and in the quest for positive evaluation. Sometimes this may not solve the root cause.’*

IDI Local Government Project Specialist

### Pervasive (urban) informality

It was reported that some interventions were not implemented on their merit and benefits to the general public.

*‘ … but how much can technocrats profiteer? In most cases, the canons of value addition and productivity are ignored. How else would one explain why SACCOS and other cash-based interventions that do not create employment are privileged over cooperatives and industrial development?’* Academic / Researcher

The ‘over urbanization’, in Uganda was in part driven by the massive reproduction of poverty and lack of a steady supply of jobs and other urban services. Slums in Kampala city had more unemployed and destitute residents than Mbarara and Gulu. This was due to the primacy of Kampala city. Overall, Kampala city had the most complicated slum narrative given its national place as the biggest administrative and business capital. Mbarara being the regional town for the south-western part of the country, had the attraction for those seeking the ‘urban dividend’ as they tried to recover from multiple vicissitudes such as crop failure and droughts. In addition, refugees from neighboring countries undergoing political turmoil resided in and around Mbarara.

*‘The affinity and means to frequently go to Mbarara city are seen as a measure of success and civility. Staying in Mbarara city sets someone apart and yet some people in Mbarara sleep in tin and cardboard houses. Luckily, during the day, all these disadvantages are invisible to the casual observer.’*

Local Government Technical staff Mbarara.

Results from community responses in Kampala were singular in expressing that *‘Kampala sibiziimbe!*’ meaning ‘in Kampala, what you see is not what you get!’ A metaphor to echo the social isolation and harsh urban economic realities that afflict most urbanites even amidst apparent plenty and a veneer of bliss reflected in the façade of urban success. On the other hand, others asserted that, ‘*Kampala Lusuuku lwaffe feena!* Loosely translated to mean, ‘*Kampala city is everyone’s hunting ground’*. It was emphasized that, Kampala is a place of the economically resourced and the socially connected. In sum, what you see isn’t what you get! It is for the agile and street smart that can evade compliance, resist, persist and manipulate public resources for private gain. There was no place that demanded ‘street survival skills’ as Kampala where poor migrants created slums by creating their own *reception centres*. As any reception centre (usually in emergencies), there was a shortage of services including sanitation. In many ways, a slum was viewed as a temporary residence to be vacated as soon as circumstances permitted. Slum inhabitants were mostly those who had not been able to move into the more ‘formal sector’ or those who, having moved, had failed to maintain this position.

*‘When I first came to Kampala, I hoped to be in a better place than this. I did not know how life works in the city. As you stay, you find your level. I could not afford to live my hopes and dreams, but I still had to stay in the city. First, with my relatives before I could be on my own. You only learn to find your level when you’re already in the thick of things. The city has its way of placing you by your means!’*

Male Rural urban migrant. Ddobi zone.

### Lack of standards: ‘to whom it may concern’ attitudes and the normalization of risk as a way of life

In the prevailing urban maze, slums offered access to cheap food, flexible terms of housing as well as makeshift sanitation facilities including free and easy disposal of human waste by *wrap and throw* polythene bags-a form of open defecation. In such a setting, WASH related challenges were overshadowed by more pressing needs manifested in income poverty. What we found does not support the thesis that ‘resistance to improvement’ is a direct result of indoctrination in the culture of poverty. While better accommodation was desirable, it couldn’t be accessed and afforded let alone sustained within the current income outlay. This is why slum housing was attractive as opposed to modern town houses as were proved by the namuwongo slum upgrading project. Thus, the effort to understand this *escape from improvement* is partly premised in its unsustainability. Slums were akin to the proverbial ‘*Shauri yako*’ a Swahili phrase serving as a caution to the fact that, everything was *‘to whom it may concern or at your own risk.’* This meant there were no standards to be upheld. Participants understood that, being in a slum meant a lowering of expectations and standards.

### ‘Slumification’ as a platform for poor urban sanitation

Urban managers and utility providers noted that, among the many illegalities in slums, were unauthorized electricity connections. Residents hooked wires on overhead service lines and trenched to avoid paying connection charges with little regard to safety concerns. Observation and community transect data showed wide spread disorder including a lack of road etiquette that was bred in a *‘slumified’* culture of disrespect for commons and communal living. It was observed and reported that, even the non- poor had adopted a slum outlook of free riding and escaping utility costs. It was reported that some property developers would excavate basements of their multistoried buildings to extract unclean water and pump it in the building where the unsuspecting public would use this water as safe. This is a manifestation of a slum life style as a chosen culture.

## Discussion

This study endeavored to assess the processes leading to the formation of slums. Previous sanitation interventions dealt with *problems in the city* (symptoms) rather than *problems of the city* (root causes). Although Uganda’s urbanization challenges are nested in Kampala, they reflect broader problems in the political economy of the country. The challenges relate to poverty, in its various dimensions, and not necessarily, the technical impossibility of transiting to a better city [33, 67]. Some of the best cities and places on earth are in the most challenging locations and terrains [68, 69]. For the most part, urban planning canons in Uganda are stood on their heads. From a functionalist perspective, slums provided an option to stay in the city for the unemployed and low-income groups [70, 71]. On the other hand, *slumified* living made life cheap and affordable. For those with the means to grab land [72], slumification provided an opportunity to establish oneself. Slum dwellers prioritized *‘a survival over dignity’ strategy* with phrases such as *‘(O)kupaanga’* (modelling life on a daily basis) *‘Kuyiiya’* (tinkering with life to see what comes your way) *‘Kweyiiya’/ ‘Kuteteenkaanya’ / ‘Okubaawo’* implying the *agitation of the setup* so that one can *get survival space*. Such metaphors negated urban planning canons including the regard for public space and goods, including sanitation. The behavioral perspective of slums relates to when and where urbanites negate the merits of responsible and organized living, planning, compliance and proper service delivery. Taken together, these findings indicate that, the affluent and deprived areas relate and intersect in sustaining undesirable outcomes for urban dwellers reflecting the need for a ‘systems thinking’ approach in addressing the urban sanitation question.

The pursuit of individual benefit heightened extractive, predatory and unsustainable actions at personal and collective levels, which in turn made provision of social services difficult. In response, people tried hard to evade taxes as a consolatory measure. It is important that, stakeholders aiming at improving urban sanitation take a broader outlook including addressing the pervasive poverty and insecure livelihoods as a means to deliver sustainable slum sanitation.

Concern over poor urban sanitation is not new [73, 74] yet sanitation among the urban poor remains deplorable [29, 75, 76]. The role of sustainable livelihoods in poverty eradication cannot be overemphasized [77–79], little wonder that, the first SDG is on eradicating poverty, emphasizing people; sustained, inclusive, sustainable economic growth and decent work [77]. For instance, SDG 11 connects to the reality that, cities can also hinder or facilitate progress towards other goals including human health, WASH and climate change as expressed in SGDs 2, 3, 6 and 13 [80]. Hunger, disease, ignorance and unstainable practices allude to the poor [81, 82]. Therefore, understanding urban poor sanitation involves analysis of the broader urban context as well as the interrelations at various levels and among actors [83].

In Uganda, beyond the suburban looks, urban centres are beset with poverty, despair, squalor and anguish [41, 84, 85] with attendant environmental problems [86, 87]. Urban poor sanitation is closely linked to persistently high rates of urbanization that outstrip the capacity of the urban economies [24, 64]. We argue that, the sanitation challenge in slums has been over simplified, in the process underestimating this complexity and deficit of services as well as coping mechanisms [24, 29, 75, 76, 88]. This is the economic rationality of slum sanitation that previous research and interventions have not addressed. While sanitation marketing focuses on creating demand for sanitation, it ignores the fundamental factors that drive and sustain this demand. Looking at demand as related to willingness, cost, availability, reliability and household attitudes [89, 90] and not linking this to affordability or the lack thereof, negates a reality of secure livelihoods which dictate uptake and sustainability of services or otherwise. At aggregate, and for the most part, the delivery of sanitation services is affected by a poor urbanization process, in the broader framework of an *informal survivalist* economy [91]. The problem of slums is linked to long-term failure by urban authorities to tackle unemployment, low productivity and the wider lack of economic opportunities outside of Kampala [22, 92, 93]. Previous sanitation interventions in slum areas, including the Kampala Urban Sanitation Project (KUSP) and the Kampala Integrated Environmental Management Programme (KIEMP) did not yield the intended results. This further makes a case for the reality that, willingness to pay, has little relevance if there is no ability to pay [94–96].

Urbanization has led to many positive developments for society, but has also contributed to many challenges [97–99]. The urbanization conundrum in Uganda is never a lack or need of a new capital city, not even an absence of urban centres. The problem is poor performance across the socio-economic spectrum with attendant regulations that are not enforced hence increasing the informal nature of urban survival and the resultant ‘*slumification’*. The  previously planned parts  of the city are gradually being eroded due to encroachment and a violation of the planning norms. Knowing that cities evolve in response to advantageous features would go a long way in informing the urbanisation agenda, where urban ecology, links city growth to resources, industry and trading opportunities [100]. One major undoing has been looking at Kampala in isolation of other parts of the country, forgetting that Kampala is intimately linked to the fortunes and resources in other parts of the country. We argue that, properly planned and evolved cities are centres of opportunity. Therefore, if cities are to be sustainable, they are neither to be ‘made’ nor ‘created’. Cities have an organic process through which they develop, survive and grow [101]. Structures that develop cities cannot be declared. This means that, Ugandan urban centres need a capacity threshold to perform their urban functions. While a city can be demanded and declared, it could all the while be *absent* in terms of services and metropolitan opportunities. It was evident that, the demands for city statuses and new administrative units are laced with vested interests including transactional politics [102]. It has been proved that, a rush to house the urban poor forced them to sale the modern houses and return to their shacks since the livelihood question was never settled [22, 75, 103]. In Uganda, there is no evidence of a slum that has disappeared by way of absorption and growth, most slums are simply displaced and relocated, Namuwongo being a case in point. Therefore, infrastructure without income for owners is a liability [22, 104].

Sustainable urbanization is a precursor to sustainable sanitation, with sustainable demand for sanitation services being driven by affordability and improved welfare. For urbanites, to ably demand, install and maintain sanitation infrastructure it must be financed. One way of doing this is through an elaborate urban *perestroika* [105, 106]. Affordability, demand and incentives to comply and improve urban sanitation are related, but different. If urban poverty is reduced [13, 107], there will be less motivation to circumvent proper sanitation due to its associated costs [108]. Better urban livelihoods create demand for improved sanitation on which marketing interventions can be built [109, 110]. The current approaches of beginning with the marketing of sanitation [111] as a means to create demand, creates short-term solutions without continuities thereafter. From a sustainable sanitation standpoint, this approach fails the sustainability test. Affordability is a major variable in sustainable sanitation services. Therefore, poverty reduction must be central in interventions to realize sustainable sanitation [112–114]. The complexity of sustainable urban sanitation needs to be more comprehensive than counting sanitation facilities in place [29, 75]. Results show that, improved sanitation cannot be the only right thing in slums. Therefore, urban synergies will go a long way in creating viable urbanization based on; value addition, sustainable urban livelihoods [115] and effective demand of higher-quality services that protect people and the entire sustainable sanitation service chain. Urban challenges are best addressed through the formalization of transactions that have financial footprints on which other efficiencies and services can thrive [115]. We argue that, there cannot be sustainability of sanitation services without effective poverty reduction [116, 117] and the attendant economic growth [118]. Policy led economic growth, development, planning and strategy are the drivers of sustainable urbanization including sustainable sanitation [119, 120].

Building effective cities requires multifaceted and well-targeted interventions. The negative externalities caused by urbanisation should be addressed through the creation of more positive externalities [116, 117]. Sanitation facility failure in slums is associated with unaffordable costs associated with sanitation access, use and maintenance [108]. Intervention failures in operation and maintenance [108] have been linked to unaffordability even when there were robust technical solutions, awareness and willingness to pay [29, 41, 75, 76]. Viable urbanization and sustainable (urban) livelihoods [115] inadvertently create and sustain the effective demand of social services that protect people, space and urban aesthetics including sanitation. The linkages between urban poverty, overcrowding and poor infrastructure manifests in form of poor sanitation [24, 29, 75, 76, 95].

We re-conceptualize sustainable urban sanitation as one that is embedded within a systemic and synergetic framework. Sustainable urban sanitation is beyond the physical availability of sanitation facilities, it is access (spatial, physical and financial) to a given facility by physical abilities and gender [76]. The core proximate factors influencing urban welfare and sustainable sanitation are; employment, income, infrastructure, housing and opportunities for urbanites. There are a number of governance [121, 122] variables that influence urban conditions, and yet these urban conditions in subtle ways influence urban governance including the levels and quality of the available sanitation choices and solutions. As such, in the quest for improved slum sanitation, we must ask; can people sustainably afford all the costs associated with maintaining desirable sanitation standards? The beginning point ought to be the availability of structures and services that appeal to the intended users. After this, use and associated costs must be affordable. What has been lacking in most previous sanitation interventions, are the ‘continuities’ with actors intervening in a vertical fashion often suddenly, independently, inadequately without addressing the root causes of existing sanitation problems.

When urban livelihoods are secure and governance systems deliver affordable municipal services, user motivation is likely to result into more compliance. Beyond enforcement, there is need for intrinsic motivations to comply with established (sanitation) norms and standards [123]. Motivation is what moves and drives people. Affordability of the available services and structures calls into question the underlying issues of population quality, income opportunities, ability to pay, employment and productivity as well as the functional capacity of the available structures at different levels to deliver urban sanitation services. We argue that, affordability of a service gradually leads to individual and collective accountability. Like all social processes, provision of sanitation is faced with ever changing situations. This means that, there should be ongoing research and development to enable the urban sanitation sector to deliver especially for the ever-changing population socio-economies [124, 125]. The urban capacity-mandate nexus calls for further interrogation as an input into planning and policy processes. An alternate (capital) city is not the answer, but revamping productivity, industry, employment and good governance that is free of incompetent and predatory managers. Sustainable urban sanitation that especially protects the urban commons is critical in regulating urban living.

Economic considerations are visible in the choices and practices by slum dwellers in as far as their means constrain, enable or modify sanitation consumption in urban areas. Therefore, this *sociology of consumption* explains what certain things mean to people and how they are ranked and used from day to day. To this extent, sanitation choices are an ongoing process. Therefore, the available incomes and, partly, absorbed values, determine how slum dwellers think about sanitation facilities within their own rationality indices. By using an economic sociology framework, we have illuminated the underpinning context that influences slum sanitation behavior and choices. This originates from within the prevailing circumstances and is a result of purposive action which is embedded within ongoing systems of socio-economic relations. While some may disagree, we posit that, the current urbanization trajectory in Uganda is unsustainable (unless checked and revisited) with an urban penalty for all; directly and indirectly. Premature and unsustainable urbanization under the guise of ‘inclusivity and a right to the city’ amounts to chaotic urban configurations that lead to a breakdown of municipal services including poor urban sanitation. Indeed, the cost of poor sanitation is immediate and severe; first to the poor, and by extension to all urban dwellers. The following lessons stand out for urban authorities and policy elites;

i) *Premature* urbanization in Uganda, is manifested in teething sanitation challenges that need a comprehensive analysis that is intimately linked to poverty.
ii) The urban poor do not need to be told about better sanitation. They already know this. They simply can’t afford it! This is the livelihood sanitation nexus.
iii) There is an under- appreciated connection between the capacity of urban authorities and the ability to deliver on their mandate. Cash strapped urban authorities cannot live up to their mandate and are prone to abuse by both the polity and technocrats.
iv) In Uganda, focus has been on addressing *problems in the city* and not *problems of the city* in the process missing the root causes while focusing on symptoms. This partly explains the culture of unsustainability.
v) An understanding of the rural- urban linkages enables sector wide interventions as opposed to quick fixes and kneejerk reactions to systemic cum- institutional urban problems.
vi) It is not the lack of awareness about sanitation but the lack of income and an urban governance deficit that shape the persistent unsustainable urban sanitation services.

### Limitations

This study is not without limitations. The basic limitation of our study is that, it is entirely qualitative. While a mixed methods study would have presented a more complete picture, even with this limitation, this study has the explanatory power of qualitative research as its major strength that is beyond comparisons and measurements. Future studies (quantitative and mixed methods) could build upon our findings.

## Conclusions

This study makes an exposition of what slum dwellers aspire to and how they maintain these aspirations that run counter to sustainable urbanization, demand and the quest for a better urban experience. In many surprising ways, for low-income earners, slums were areas which were *sought after* rather than habitats to be shunned. Better choices were hoped for later in life. For the newcomers, economic survival trumped proper sanitation as the overriding consideration. Sustainable urban sanitation cannot be the only right thing happening in such settings. Before urban sanitation improves, a lot has to change in the urban configuration. The idea of a single intervention solving every sanitation problem in slums is erroneous. Addressing poor sanitation in slums calls for the reframing of the urbanization question and processes currently being undertaken. Sanitation interventions need to be practical and relevant to the population, progressive, successive, accessible, inclusive, consistent, affordable, innovative and responsive to the needs and realities on ground. Improving urban sanitation requires broader and multi-faceted interventions that concurrently address poverty, unemployment, livelihood and governance deficits. Urban interventions including policies need to be linked to creating opportunities for income growth needed, so as to avoid poverty, thereby promoting sustainable urbanization. Development should drive urbanization and not the other way round.

## Supplementary Information

**Additional file 1 Supplementary file 1** Research instruments.

## Data Availability

The research data shall be available for sharing upon reasonable request. All inquiries and data requests should be sent to the first author JNK at: nkjapheth@yahoo.co.uk

## Abbreviations

SSSC: Sustainable sanitation service chain
IDI: Id-depth interview
IRB: Institutional review board
UNCST: Uganda national council of science and technology
