# Supplementary material for: Re-conceptualizing sustainable urban sanitation in Uganda: why the roots of ‘Slumification’ must be dealt with
Source: BMC Public Health. 2021 May 26;21:992. doi: 10.1186/s12889-021-11029-8 (PMC8157622; doi:10.1186/s12889-021-11029-8)
Supplement: Supplementary file 1 — Additional file 1 Supplementary file 1 Research instruments. [file 12889_2021_11029_MOESM1_ESM.docx]

**Focus Group Discussion (FGD) Guide for analyzing Livelihood sanitation linkages in slums (landlords, tenants- women, men and youth)**

Please *u*se the notebook to make notes, record group category, district, zone name and location

1. **Background information**
2. On average, how long do people stay in one house before they move out/shift?
3. What are the major and common reasons why people move from one house to another? What is the average family size here?
4. Is the presence/state of a latrine a factor in the choosing of a house? (Ask plausible reasons).
5. **Livelihoods**
6. What is the major source of income in this area? For men, women and youths (18-35)?
7. What is the average; daily, weekly and monthly income in this area?
8. On what do people mostly spend their income? Probe for consumption, investment, health care etc. Try and have a rank of this expenditure
9. **Sanitation status**
10. On average, how many households use a latrine facility in this zone?
11. On average, how many stances does a latrine facility in this zone have?
12. Do stance/latrine users know each other?
13. How often are shared latrines cleaned? Capture consensus from the group.
14. How often should shares latrines be cleaned?
15. Who is responsible for cleaning shared latrines here? Captures the consensus caretaker and why?
16. Of the latrine users; is there anyone likely to influence cleaning of the latrine? Probe for this person …
17. Who should clean and maintain shared latrines? Capture by categorization e.g. income or any other grouping…
18. Are landlords/house owners, local leaders and tenants bothered about dirty latrines in this area?
19. What types of latrines do people mostly use in this zone/area? Probe for shared or private facilities…and why?
20. Are sanitation facilities in this area, mostly dirty or clean? Which types are mostly clean and which are mostly dirty?
21. What are the main causes of sanitation facilities being dirty?
22. Which people make sanitation facilities dirty/don’t mind dirty sanitation facilities?
23. What problems /major obstacles are encountered in the process of cleaning shared latrines?
24. What is the role of income/ employment/ assets and welfare in keeping latrines clean?
25. Are dirty latrines a priority issue to be addressed in this zone? (Categorize and probe different responses/perspectives
26. **The role of livelihoods in sanitation status and maintenance**
27. Are there differences in sanitation;
28. Access
29. Demand
30. Use
31. Maintenance

Among different income groups in this area? If yes, elaborate these differences.

1. Are there (income) groups of people are interested in keeping sanitation facilities clean? Which groups are these?
2. Are there any actions that have been undertaken to overcome latrine cleaning and maintenance problems in this area?
3. What can; landlords/house owners, tenants and local leaders do to improve latrine cleanliness?
4. Do you have any other suggestions and comments on how shared latrines can be kept clean and well maintained?

**Key informant interview guide –KII for Local leaders, landlords and technical staff**

Please *u*se the notebook to make notes, record group category, district, zone name and location

1. What are the major sanitation challenges in this slum (area)? /Why are shared latrines dirty?
2. Which groups of people least afford sustainable sanitations services?
3. How are income and sanitation status related in this area?
4. What are the income related obstacles/barriers to sanitation in this area?
5. Which categories of people are most likely to demand and afford sustainable sanitation services?
6. Would you say that the presence and state of a latrine is a factor for slum dwellers when choosing a house? What initiatives are you aware of that try to keep shared latrines clean in slums?
7. What are some of the challenges in cleaning shared latrines?
8. Who can influence people to clean and maintain shared latrines in slums?
9. How can people be encouraged to keep shared latrines clean?
10. Are there variations among men, women and youth in using and keeping shared latrines clean (Benefits and risks of gender)
11. Is there a way, shared latrine status, use and cleaning can be linked to income and common interests and groups/joint ventures?
12. How can latrine presence and status be a factor for slum dwellers when choosing a house?
13. Any other suggestions and comments on how shared latrines can be kept clean and well maintained?

**Thank you very much for your time and cooperation**

**In-depth interview –IDI Guide for tenants**

1. What are the major sanitation challenges in this slum (area)? /Why are shared latrines dirty?
2. Which groups of people least afford sustainable sanitations services?
3. How are income and sanitation status related in this area?
4. What are the income related obstacles/barriers to sanitation in this area?
5. Which categories of people are most likely to demand and afford sustainable sanitation services?
6. Would you say that the presence and state of a latrine is a factor for slum dwellers when choosing a house? What initiatives are you aware of that try to keep shared latrines clean in slums?
7. What are some of the challenges in cleaning shared latrines?
8. Who can influence people to clean and maintain shared latrines in slums?
9. How can people be encouraged to keep shared latrines clean?
10. Are there variations among men, women and youth in using and keeping shared latrines clean (Benefits and risks of gender)
11. Is there a way, shared latrine status, use and cleaning can be linked to income and common interests and groups/joint ventures?
12. How can latrine presence and status be a factor for slum dwellers when choosing a house?
13. Any other suggestions and comments on how shared latrines can be kept clean and well maintained?

**Thank you very much for your time and cooperation**
